# Photodegradation of organic pollutants RhB dye using UV simulated sunlight on ceria based TiO_2_ nanomaterials for antibacterial applications

**DOI:** 10.1038/srep38064

**Published:** 2016-11-30

**Authors:** Kaviyarasu Kasinathan, John Kennedy, Manikandan Elayaperumal, Mohamed Henini, Maaza Malik

**Affiliations:** 1 UNESCO-UNISA Africa Chair in Nanosciences/Nanotechnology Laboratories, College of Graduate Studies, University of South Africa (UNISA), Muckleneuk Ridge, P O Box 392, Pretoria, South Africa; 2 Nanosciences African network (NANOAFNET), Materials Research Group (MRG), iThemba LABS-National Research Foundation (NRF), 1 Old Faure Road, 7129, P O Box 722, Somerset West, Western Cape Province, South Africa; 3 National Isotope Centre, GNS Science, PO Box 31312, Lower Hutt 5010, New Zealand; 4 Department of Physics, TVUAC, Thennangur, Thiruvalluvar University, Vellore, India; 5 School of Physics and Astronomy, The University of Nottingham, University Park, Nottingham, Nottingham NG7 2RD, United Kingdom

## Abstract

To photo-catalytically degrade RhB dye using solar irradiation, CeO_2_ doped TiO_2_ nanocomposites were synthesized hydrothermally at 700 °C for 9 hrs. All emission spectra showed a prominent band centered at 442 nm that was attributed to oxygen related defects in the CeO_2_-TiO_2_ nanocrystals. Two sharp absorption bands at 1418 cm^−1^ and 3323 cm^−1^ were attributed to the deformation and stretching vibration, and bending vibration of the OH group of water physisorbed to TiO_2_, respectively. The photocatalytic activities of Ce-TiO_2_ nanocrystals were investigated through the degradation of RhB under UV and UV+ visible light over a period of 8 hrs. After 8 hrs, the most intense absorption peak at 579 nm disappeared under the highest photocatalytic activity and 99.89% of RhB degraded under solar irradiation. Visible light-activated TiO_2_ could be prepared from metal-ion incorporation, reduction of TiO_2_, non-metal doping or sensitizing of TiO_2_ using dyes. Studying the antibacterial activity of Ce-TiO_2_ nanocrystals against *E. coli* revealed significant activity when 10 μg was used, suggesting that it can be used as an antibacterial agent. Its effectiveness is likely related to its strong oxidation activity and superhydrophilicity. This study also discusses the mechanism of heterogeneous photocatalysis in the presence of TiO_2_.

Solar energy is uniquely poised to solve major energy and environmental challenges that are being faced by humankind. As such, it is important to develop a suitable environmentally-friendly technology that permits the full range of the solar spectrum to be used for simultaneously solving energy and environmental challenges. It has been proposed that it is possible to address these challenges using nanocomposite materials that are capable of solar photocatalytic conversion^1^,^2^. Nanocomposite materials have a mixture of different chemical compositions and have received wide interest from fundamental and applied science researchers. The physical properties of these materials can be combined to produce materials that have desirable characteristics. Optical or biological characteristics can change with decreasing particle sizes, which is a major reason for interest in nanocomposite materials^3^. For example, metal oxide nanocomposites have excellent physical properties, such as high hardness and melting points, low densities and coefficients of thermal expansion, high thermal conductivities, good chemical stabilities. They also have improved mechanical properties, such as higher specific strengths, better wear resistance and specific modulus, and have wide potential for various industrial fields^4^,^5^.

Metal oxide semiconductor photocatalysts (MOSPs) offer a number of opportunities for enhancing energy efficiency and reducing environmental pollution through minimizing carbon footprints^6^,^7^. Titanium oxide (TiO_2_) is a MOSP of particular interest because of its unique properties, such as being a wide forbidden energy band gap semiconductor, its non-toxicity to living organisms, stability in water, and strong photocatalytic properties when its crystal grain size is reduced to tens of nanometers^8^. The strong redox power of photo-excited TiO_2_ was realized with the discovery of Honda-Fujishima effect in 1972, where Fujishima *et al*., reported the photo-induced decomposition of water on TiO_2_ electrodes^9^. TiO_2_ acts as a photocatalyst under ultraviolet light, but this only contributes to approximately 2–5% of total solar power, so the photovoltaic or photocatalytic activity of TiO_2_ is limited^10^,^11^. Another major limitation of TiO_2_ is massive photogenerated electron−hole recombination, which limits the efficiency of the photocatalyst. CeO_2_, on the other hand, has been shown to be a promising candidate because of its desirable band edge positions and it has been successfully used in a number of photocatalytic processes, such as detoxification and hydrogen production. The redox shift between Ce^4+^ and Ce^3+^ can create a high capacity for the system to store or release oxygen under oxidizing or reducing conditions^12^,^13^,^14^,^15^. Many studies have reported that CeO_2_-TiO_2_ systems have enhanced properties under UV solar irradiation. In particular, Zhang *et al*., found that cerium doping could prohibit the recombination of photogenerated electron-hole pairs^16^. Yan *et al*. reported the preparation of Ce-doped titania through a sol-gel auto-ignition process that had strong absorption in the UV-vis range and a red-shift in its band gap transition^17^,^18^. It has been proposed that doping the base photocatalyst with impurities is best way to enhance the utilization of solar energy and to inhibit the recombination of photogenerated e^−^ - h^+^ pairs. Xiao *et al*., synthesized Ce-doped TiO_2_ mesoporous nanofibers using collagen fibers as a biotemplate and showed that RhB dye on the Ce_0.03_/TiO_2_ nanofibers degraded by 99.59% over 80 min under visible light, much higher than undoped TiO_2_ nanofibers or the commercial product Degussa P25^19^,^20^. In this study we report a detailed investigation of CeO_2_-doped TiO_2_ nanocrystals and their structural properties, crystallinities, phase transformations, morphologies, and photocatalytic and band gap engineering. Photocatalytic activities were examined using Rhodamine B (RhB; 99.95% purity) as a model impurity under solar irradiation (λ > 365 nm). For the first time we also present the antibacterial activity of CeO_2_-doped TiO_2_ nanocrystals that were prepared using a hydrothermal method and the mechanism responsible for the synergistic effects of CeO_2_-doped TiO_2_ nanocrystals is also discussed in detail.

## Experimental section

### Chemicals & typical synthetic process for CeO_2_ - TiO_2_ nanocomposites

All chemical reagents (analytical grade) were used as received (E-Merck 99.99%) without further purification. To synthesize mixed cerium-titanium oxide nanocomposites, 0.1 Mol % of cerium (III) nitrate hexahydrate [Ce(NO_3_)_3_·6H_2_O] and 0.1 Mol % titanium (IV) nitrate [Ti(NO_3_)_4_·4H_2_O] were combined with distilled water and stirred thoroughly using a magnetic stirrer. For hydrothermal synthesis, nearly 0.5 Mol % cetyltriethylammonium bromide (CTAB) [C_19_H_42_BrN] solution was prepared and added dropwise to the cerium-titanium solution that was vigorously stirred, the color of the solution turned yellowish dark pink in 5 min, indicating the beginning of the reaction. Then the color of the solution became deeper and deeper with the increase in reaction time till it became completely dark, demonstrating the formation of CeO_2_-TiO_2_ nanoparticles. The homogeneous mixture was loaded into a 250 mL Teflon-coated stainless lined autoclave, which was then filled with distilled water to 70% of the total volume. Then the above solution was transferred to Teflon lined autoclave at 240 °C in a micro oven for 24 hrs. The synthesized nanoparticle was annealed at 700 °C in a microprocessor controlled single zone furnace for 9 hrs. After the reaction was completed, the controlled furnace was cooled to room temperature and the resultant solid products were collected, washed several times with absolute ethanol and distilled water and then dried at 140 °C for 9 hrs. Finally, the obtained nanoparticles of CeO_2_-TiO_2_ were used for different characterization studies.

### Sample characterization

High-resolution transmission electron microscopy (HRTEM) measurements were made on a HITACHI H-8100 electron microscope (Hitachi, Tokyo, Japan) with an accelerating voltage of 200 kV. The samples for HRTEM characterization were prepared by placing a drop of colloidal solution on carbon-coated copper grids and drying at room temperature. The elemental compositions were determined using selected area electron diffraction (SAED) (IH-300X). Optical absorption was measured using a Varien Cary 5E spectrophotometer with excitation wavelengths ranging from 350–700 nm. The confirmation of CeO_2_-TiO_2_ nanoparticles was performed using a UV-vis spectrophotometer. Aliquots (5 mL) of the suspension were measured to determine the surface plasmon resonance absorption maxima using distilled water as reference. The functional groups in the CeO_2_-TiO_2_ nanocrystals were evaluated using Fourier transform infrared spectroscopy (FTIR) and the spectra were recorded with a Brukker IFS (66 V) spectrometer. X-ray photoelectron spectroscopy (XPS) measurements were made using an ESCALAB 250 photoelectron spectrometer (Thermo-VG Scientific, USA) with Al Kα (1486.6 eV) as the X-ray source. Photoluminescence (PL) measurements of the as-synthesized products were carried out using an F-4500 KIMON fluorescence spectrophotometer at room temperature with a Xe lamp as the excitation source. The excitation wavelength used was 397 nm.

### Dye adsorption testing

The dye activity performances of the synthesized nanocomposites were tested for their ability to remove textile dye in an aqueous solution. RhB, a carcinogenic textile dye, was used as the organic impurity model. The RhB adsorption experiment was conducted in 15 mL capped glass tubes containing 5 mL of RhB solution (15 mg/g) and 15 mg of the synthesized composite nanocrystals. The sample-containing glass tubes were then placed into a Certomat WR-Braun Biotech International temperature-controlled water bath shaker at a constant agitation speed (120 rpm) and 25 ± 1 °C. After some time the glass tubes were removed. The RhB filtrate was separated from the solid material. Absorbance of the filtrate was then measured using a U-3501 Shimadzu UV-vis-NIR spectrophotometer at the wavelength maximum of RhB (λmax = 500 nm). The concentration of RhB that remained in the sample solution was calculated from a calibration curve. The percentage of RhB adsorption expressed as shown in Equation: % Adsorption = ((C_0_ − Ct)/C_0_) × 100%, where C_0_ is the initial concentration of RhB dye (mg/L) and Ct is the concentration of RhB dye remaining at time (t) (mg/L).

### Photocatalytic performance test

The photocatalytic activity of each sample was studied from the degradation of RhB under UV (λ < 400 nm), visible (λ > 400 nm), and UV + visible light. The UV and visible irradiances at the reactor surface were 0.15 W/m^2^ (Philips 15 W/G15 T8, Holland) and 14.5 W/m^2^ (Philips 18 W/54 1M7 India), respectively. The catalytic material loadings for the experiments were 0.5 g/L and the average reactor temperature was maintained at 35 °C. The solutions were kept in the dark for two hrs to achieve adsorption−desorption equilibrium. The experiments were carried out by simultaneous exposure of the catalysts, each of which had 30 mL of RhB (1 mM) that was being stirred. The catalyst-loaded RhB solutions were illuminated under UV, visible, and UV + visible light for 60 min and sampling was performed at 15-min intervals. At specific time intervals the photo-reacted solutions of the centrifuged samples were analyzed by recording variations in the absorption band maximum (664 nm) using a UV−visible spectrophotometer (Shimadzu 1700, Japan).

### Antibacterial activity performance

The antibacterial activities were evaluated against *E. coli*, a Gram-positive bacterium. *E. coli* was obtained in frozen form from the American Type Culture Collection. The bacteria were thawed on ice for 20 minutes before being placed on an agar plate. The plate was then dried before incubation for 16 hrs in a standard cell culture environment (37 °C, 5% CO_2_, and 95% air). A single colony of *E. coli* was selected using a 10-μL loop and inoculated into a centrifuge tube containing 5 mL of cryptic soy broth. Bacteria in the centrifuge tube were then incubated at 37 °C under agitation at 200 rpm for another 16 hrs. At that point, the bacteria solution was diluted in cryptic soy broth to an optical density of 0.52 at 600 nm using a microplate reader. According to the standard curve correlating bacteria number with optical density, this value was equivalent to 5 × 10^6^ cells/mL. The cells were further diluted in cryptic soy broth to 5 × 10^4^ cells/mL before being added to a new centrifuge tube at 5 mL/tubes. Concentrated Ce-TiO_2_ nanoparticles in solution were added to bacteria tubes at different doses (0.08 mM (low dose), 0.15 mM (medium dose), and 0.3 mM (high dose)). A tube of bacteria without nanoparticles served as a control. Bacteria were then incubated under agitation for four hours, 12 hrs, and 24 hrs, before a 200-μL bacteria solution was transferred to a 100-well plate for optical density readings at 600 nm using a microplate reader.

## Results and Discussion

**Figure 1:**
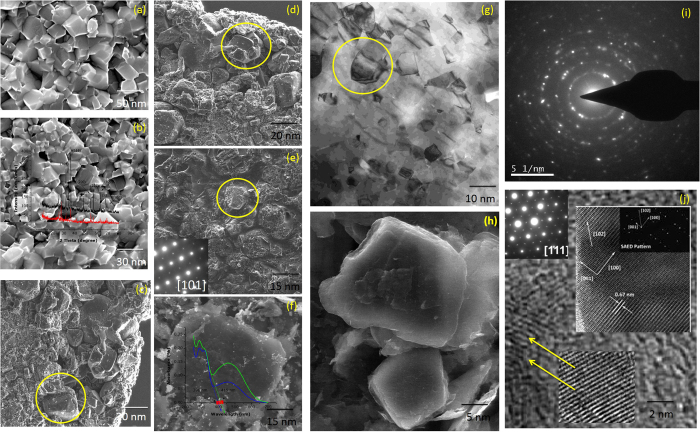
(a–j) High Resolution Transmission Electron Microscopy images of CeO_2_ doped TiO_2_ nanocomposites were synthesized hydrothermally at 700 °C for 9 hrs.

High-resolution TEM images of the ceria-doped TiO_2_ nanocomposites are shown in Fig. 1(a–j). It is apparent that the nanocomposites consist of a large quantity of spherical-like cubic nanocrystals with a narrow size distribution. Upon addition of 0.5 Mol% CTAB and calcination for 3 hrs, aggregation of CeO_2_ nanocrystals occurred. These nanocrystals could not be clearly distinguished from each other^21^,^22^,^23^. When the calcination treatment was prolonged to 5 hrs, the morphology became more regular and the nanocrystals displayed a suitable morphology, with the product being dispersive, as shown in Fig. 1(a–e), when the calcination time was extended to 7 hrs, it can be seen from Fig. 1(f–h) that the nanocrystals were overburnt and had irregular forms. Aggregation of spherical particles occurred along with the formation of crystals; however, the nanocrystals were difficult to separate. This may have occurred because the long calcination time caused collapse of the morphologies. In the bright field image, all of the synthesized samples appeared to consist of many semi-aggregated round particles. The average particle size of ceria-doped TiO_2_ nanocomposites, as shown in Fig. 1(b–d), was estimated to range from 15–30 nm and Ce-Ti particle sizes (Fig. 1(g–h)) ranged from 5–10 nm. The HRTEM image also shows individual CeO_2_ nanocrystals with good crystallinity and clear lattice fringes, highlighting that both materials had regular spherical shapes and narrow size distributions. The composites prepared hydrothermally consisted of 5–10 nm particles that were agglomerated to form porous, irregular networks and consisted of monodisperse CeO_2_ nanoparticles, as shown in the HRTEM images.

The HRTEM micrographs and selected area diffraction patterns of the Ce-TiO_2_ nanocrystals are shown in Fig. 1(i,j). The diffraction peaks were very diffuse, suggesting that the texture was polycrystalline with small grain sizes^24^,^25^,^26^. Therefore, the distributed pore sizes and mean pore diameters obtained from N_2_ adsorption–desorption analyses would represent the values for the whole crystal network. It should be noted that the particle sizes of CeO_2_ obtained in presence of nanotitania were small, even in the absence of additional stabilizing agents, and this included the CeO_2_ nanoparticles deposited on the TiO_2_ lattice. Since Ti is a known catalyst for redox reactions, this suggests that Ti catalyzed the formation of CeO_2_. Because the surfaces of TiO_2_ nanoparticles were not completely covered with CeO_2_, it is possible that Ti and Ce sites featuring different ligands like (–Ce) or (–COOH) could be used for optoelectronic devices. The total Ti content of the sample was 4.8 wt%, corresponding to a Ce content of 5.2 wt%. The monocrystalline ceria also showed a layer-by-layer structure (Fig. 1(j)), which was consistent with results from the XRD pattern provided in the Supplementary Information Figure S1. The selected area diffraction pattern of the nanocrystals in the bottom of Fig. 1(j) confirmed that Ce was monocrystalline along the [111] direction (inset Fig. 1(j)). The selected area diffraction pattern with diffraction rings was calculated and confirmed the presence of ceria-doped TiO_2_ nanocrystals in Fig. 1(i).

**Figure 2:**
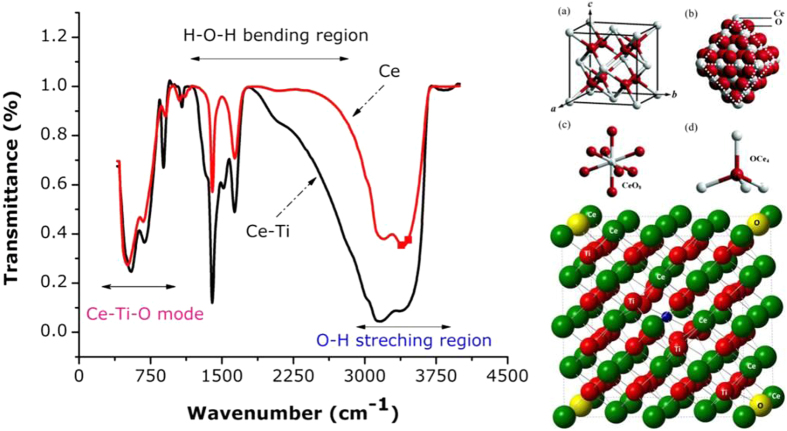
Fourier Transmittance Infrared spectrum CeO_2_ doped TiO_2_ nanocomposites were synthesized hydrothermally at 700 °C for 9 hrs.

The elucidation of structural features using FTIR is shown in Fig. 2. In the FTIR spectra of ceria-doped TiO_2_ nanocrystals and TiO_2_ treated with a coupling agent had absorption peaks at 3426 cm^−1^ and 1626 cm^−1^ and the spectra were analyzed between 400 cm^−1^ and 4500 cm^−1^. The nanocrystals exhibited bands assigned to (Ce-Ti-O) and Ti-O near 544 cm^−1^ and 798 cm^−1^ that were from the longitudinal optical mode (LOM). The red shift of the LO mode of amorphous TiO_2_ from 870 cm^−1^ to 798 cm^−1^ was a consequence of the presence of CeO_2_ nanograins^27^,^28^,^29^,^30^. The intensity of the band assigned to the LO mode of the amorphous TiO_2_ phase increased with increasing TiO_2_ content. The surface hydroxyl (OH) groups of Ce-TiO_2_ nanocrystals have been recognized to play an important role in photocatalytic behavior. This is because they adsorb reactant molecules and directly participate in the reaction mechanism through trapping photogenerated holes to form hydroxyl radicals. There are limited reports identifying OH groups on the surface of Ce-O-Ti^31^,^32^. We suggest that the two sharp absorption bands at 1418 cm^−1^ and 3323 cm^−1^ were from deformation and stretching, and the bending vibration of the OH group of water physisorbed to TiO_2_, respectively, while the shoulder at 3238 cm^−1^ from Ti-OH bonds can be ascribed to the strong interaction between Ti ions and OH groups. The chemical bonding of the nanopowder was scrutinized by correlating the peaks in the spectrum to the vibration or stretching of various functional groups. The broad band peaks located at 3400 cm^−1^ & 1600 cm^−1^, which were observable in as-prepared nanopowders, were attributed to the stretching and bending vibrations, respectively, of O-H groups from absorbed water molecules^33^. These two broad bands were not detected in the spectra of calcined powders because of dehydration during calcination. The absorption band at 1380 cm^−1^ that can be attributed to the existence of nitrate groups was only observed in the as-synthesized sample, suggesting that the complete removal of this functional group can be achieved after calcination.

For the nanosamples calcined at 700 °C over 9 hrs, the appearance of new bands below 1000 cm^−1^ were observed. These peaks are from the stretching modes of Ce-O and Ti-O nanocrystals according to Zhou *et al*.^34^ & Phoka *et al*.^35^. In this study, there was little absorption at the same wavelengths in the spectra, indicating that -OH groups disappeared when nano-TiO_2_ was treated using a coupling agent. The reasons for this were chemical reactions between -OH groups of TiO_2_ and the Ce-Ti-OH group of the coupling agent in Ce-TiO_2_ nanocrystals. The absorption peaks at 512 cm^−1^ in both spectra were the character absorption peaks for Ce-O-Ti vibrations, which showed that the coupling agent only reacted with -OH groups and not TiO_2_. This shows that the structure and composition of TiO_2_ did not change after treatment with the coupling agent. Normally the defect structure of Ce-Ti-O formed by oxygen vacancies favors the adsorption of water on the surface and then dissociation of water into hydroxyl groups and protons. This dissociation behavior leads to charged surfaces on the Ce-TiO_2_ nanocrystals because of the loss or gain of protons and complexation reactions of surface hydroxyl groups.

**Figure 3:**
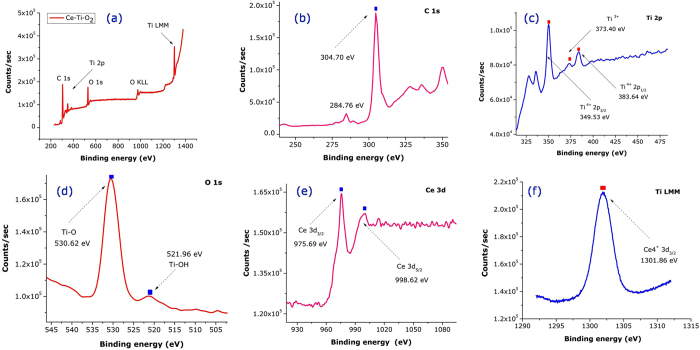
(a–f) X-ray Photoelectron Spectroscopy images of CeO_2_ doped TiO_2_ nanocomposites were synthesized hydrothermally at 700 °C for 9 hrs.

XPS measurements were carried out to understand changes in surface chemical bonding and the electronic valence band positions of Ti and Ce in the TiO_2_ and 0.5% CeTi nanostructures. Figure 3(a) presents the overall XPS spectrum containing peaks for Ce, Ti, O, and C. The XPS spectrum of 0.5% CeTi shows a binding energy peak for Ce 3d along with the Ti 2p and O 1 s orbitals. The peak at 284.76 & 304.70 eV signals the presence of elemental carbon as a reference. In Fig. 3(e) it is clear that the dominant spin–orbit doublet of 3d CeO_2_ was from Ce^3+^ and the smaller doublet was from Ce^4+^ at higher binding energies^36^. From the deconvolution result, we also observed additional satellite peaks for Ce^3+^, but not for Ce^4+^. It has previously been reported that rare earth compounds with unpaired electrons can produce an extra satellite peak from photoelectron energy gain and loss^37^. Ce^3+^ has one 4f electron, but Ce^4+^ has none. Accordingly, the extra electron can produce additional electronic transitions and give rise to the satellite peaks^38^. The XPS spectra of the Ti 2p region of TiO_2_ and 0.5% CeTi are shown in Fig. 3(c). The Ti 2p peak in TiO_2_ appears as a single, well-defined, spin-split (3.2 eV) doublet that was assigned to Ti 2p_1/2_ and Ti 2p_3/2_, which corresponded to Ti^4+^ in the tetragonal structure.

The binding energies of the peaks were found to be 383.64 eV for Ti 2p_1/2_ and 349.53 eV for Ti 2p_3/2_, which were in agreement with the binding energies of TiO_2_ previously reported^39^. This shifting corresponded to an intermediate oxidation state of Ti from tetra- to trivalent. The Ti 2p_1/2_ region was fitted into two peaks of Ti^3+^ and Ti^4+^ (373.40 and 349.53 eV). Figure 3(c) shows the binding states of oxygen in TiO_2_, with the O 1 s XPS peak fitted to three deconvoluted peaks. These peaks appeared at 349.53, 373.40, and 383.64 eV. The peak at 349.53 eV is typically from the O_2_^−^ ion in the TiO_2_ crystal structure. The binding energy peaks at 349.53, 373.40 and 383.64 eV were assigned to Ti-OH, Ti-O and Ti-O-Ce, respectively. The binding energy of O 1 s for surface oxygen shifted from 530.62 to 521.96 eV. This O 1 s peak shift suggests that Ti and Ce chemically interact with each other in the CeO_2_-doped TiO_2_ system. Figure 3(d) shows the Ce 3d peaks, which confirmed the doping of Ti into CeO_2_ nanomaterials. The peak intensity was not strong because of the low dopant concentration. The core level Ce 3d_3/2_ and Ce 3d_5/2_ peaks were observed at binding energies of 530.62 and 521.96 eV, respectively. The main XPS peak for O1s appeared at 530.62 eV. A small hump was also noticeable at 521.96 eV that was assigned to the adsorbed oxygen in CeO_2_. The peak position of oxygen was slightly shifted because of the incorporation of Ti ions into CeO_2_ when the peaks were compared with pure CeO_2_ nanocrystals. From the deconvolution results we found another small, intense peak for O 1 s at 505.32 eV that was not from the presence of Ce^3+^ or Ce^4+^, but was probably from the presence of OH on the sample surface. The XPS analyses confirmed the doping of Ce and successive formation of Ce-doped TiO_2_ nanosamples. Figure 3(e) shows the Ce core level spectrum of CeO_2_ from 930–1080 eV. From the XPS spectrum it is evident that the 3+ and 4+ valence states were present because of the non-stoichiometric nature of the material^40^,^41^. The main intense peaks of Ce^3+^ 3d_3/2_ and Ce^3+^ 3d_5/2_ were located at binding energies of 975.69 and 998.62 eV, respectively. The other peaks for Ce^4+^ 3d_3/2_ and Ce^4+^ 3d_5/2_ appeared at 1301.86 eV.

**Figure 4:**
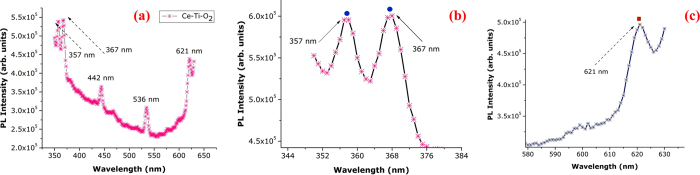
(a–c) Photoluminescence images of Ce-Ti-O_2_ nanocrystals by hydrothermally at 700 °C for 9 hrs.

The room temperature PL spectra of ceria-doped TiO_2_ nanocrystals using a 30 W laser control are shown in Fig. 4(a–c). At higher temperatures the PL spectra peaked at 600 nm (1.88 eV) in the red region and no additional changes in the spectral shapes or peak positions occurred to room temperature (RT)^42^,^43^,^44^. Two optical edge centers (green and red emission bands) of CeO_2_ and TiO_2_ nanocrystals were investigated using PL at RT. The emission spectra of CeO_2_-doped TiO_2_ nanocrystals with different Ce:Ti mole ratios are presented in Fig. 4(a–c). The emission spectra of ceria-doped TiO_2_ nanocomposition were characterized by three peaks near 357, 367 and 442 nm. All emission spectra (λ_exc_ = 320 nm) showed a prominent band centered at 442 nm that was attributed to oxygen-related defects in the CeO_2_-TiO_2_ nanoregime. The dominant defects in CeO_2_ are oxygen vacancies^45^,^46^,^47^,^48^. The intensity of the band at 442 nm increased with increasing CeO_2_ content in the CeO_2_-doped TiO_2_ nanocomposites. The formation of these compounds is likely the reason for optimal PL characteristics. Transmission from the nanocomposites was observed to be the lowest, while higher transmission occurred from the nanocrystals. However, it has been observed that the transparency of the nanocrystals adversely affected their PL response, suggesting the least transparent nanocrystals exhibit the highest PL intensities. The two prominent peaks at 357 nm and 367 nm are characteristic of the Ce^3+^ state. The XPS studies discussed earlier confirmed the presence of Ce^4+^ and Ce^3+^ states.

In this work we have reported the first dark blue nanocomposites that become increasingly transparent as the annealing temperature increased. Based on the observed shape evolutions, a possible formation mechanism for CeO_2_-doped TiO_2_ nanocrystals produced hydrothermally is presented for the first time. However, trivalent Ce has only one electron in the 4*f* state. The ground state of Ce^3+^ is split into 2F_7/2_ and 2F_5/2_. The next highest state originates from the 5*d* state and 4*f*–5*d* transitions are parity allowed. Unlike the 4*f* electron with the shielding effect of the outer shell 6 *s* and 5*p* electrons, the shift of the 5*d*, and hence the *d–f* emission band of the Ce^3+^ ion, is heavily dependent on the local crystal field surrounding the Ce^3+^ ion. Thus, the emission wavelength of Ce^3+^ is very sensitive to the crystallographic environment. Decreased PL intensity of the CeO_2_-doped TiO_2_ nanocomposites was observed when the cerium concentration increased because of increasing intra-ionic and non-radiative relaxation among Ce ions. When splitting of the 5*d* state is large and the energy difference between the lowest 5*d* sublevel and the ground state 4*f* configuration of Ce^3+^ is small, a red shift of emissions takes place. To the best of our knowledge, we have shown the first emission shift when TiO_2_ concentrations increase. When the TiO_2_ concentration is high, the unit cell of CeO_2_ becomes larger. This leads to the lowest sublevel of the 5*d* state of Ce^3+^ decreasing in energy because of a stronger crystal field when the TiO_2_ concentration is high. We observed a red shift of emissions from 536 nm to 621 nm.

**Figure 5:**
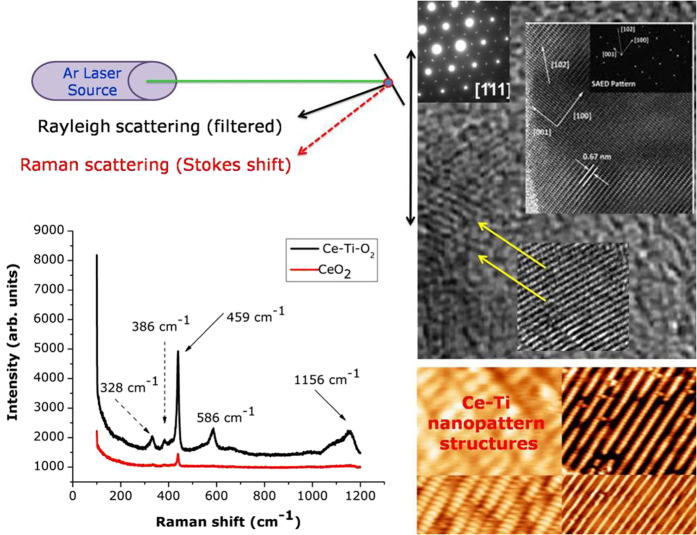
Raman spectrum of Ce-Ti-O_2_ nanocrystals.

Raman spectroscopy of polycrystalline materials has a higher sensitivity for characterizing the crystal phase of oxide nanocomposites than HRTEM. The Raman scattering profiles provided further evidence that the rutile phase was the major crystalline structure in all three targets. Figure 5 shows the Raman spectra for the TiO_2_ nanocrystals and the sharp peak at 328 cm^−1^ confirmed the existence of anatase TiO_2_. However, this peak value deviated from the theoretical value of 244 cm^−1^ and this blue shift may be caused by non-stoichiometry or the small size effect^49^,^50^,^51^. Raman scattering also showed two strong peaks at 459 cm^−1^ and 586 cm^−1^ representing the rutile E_g_ and rutile A_1g_ phases, respectively^52^,^53^. Since only the O atoms move, the vibrational mode was almost independent of the ionic mass of cerium. The broad peak in the Raman spectrum was mainly from stretching vibrations of CeO_2_ nanoparticles, which is the building block for the formation of nanocrystals. A weak peak at 1156 cm^−1^ was observed in the TiO_2_ spectrum that showed the anatase phase was present. More explanation about the rutile and anatase phases from the XRD pattern is discussed in the supplementary information (SI) and the existence of a small amount of anatase phase TiO_2_ is apparent in the nanocomposites. The Ce-TiO_2_ nanocrystals had a very strong signal for the anatase phase; the doping of Ce-TiO_2_ nanocrystals may enhance the growth of anatase TiO_2_.

**Figure 6:**
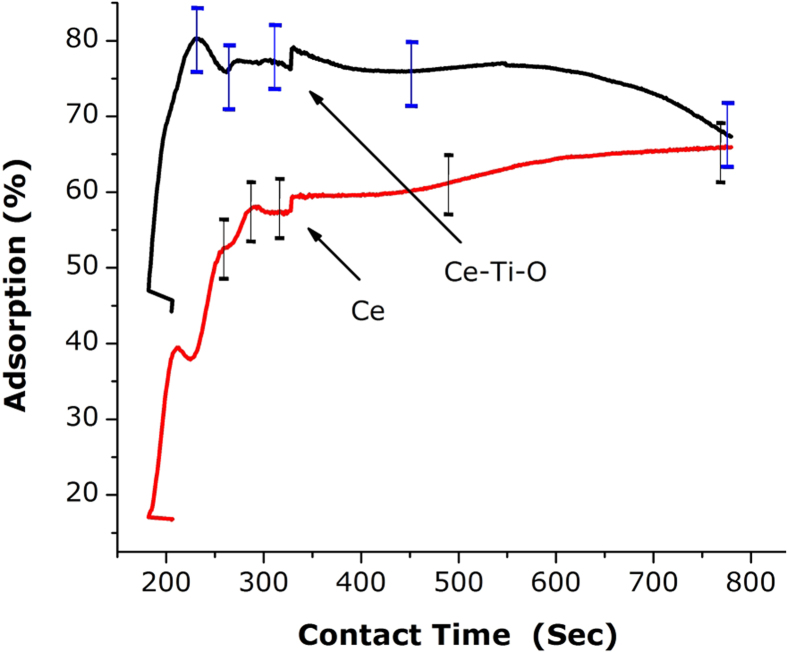
Effect of contact time on RhB adsorption Ce-Ti-O.

The performance of the as-synthesized nanosamples was tested. Pure Ce and doped Ce-TiO_2_ nanosamples showed strong absorptive capacity for RhB dye, and because of this RhB was used as a model pollutant^54^,^55^,^56^,^57^,^58^,^59^,^60^. Figure 6 shows the time-dependent RhB adsorption by Ce and Ce-TiO_2_ at room temperature. The removal efficiencies of both nanosamples showed similar trends, where adsorption rates increased rapidly over the first 3 min and then stayed steady as time passed. The equilibrium times for pure Ce and Ce-TiO_2_ were found to be 3 min and 5 min, respectively. The ability of Ce-Ti-O to adsorb the RhB dye was higher than that for pure Ce, which is apparent from the curve. The smaller particle size of CeTi than that of pure Ti may play an important role in the adsorption process^61^,^62^,^63^,^64^. This is likely because smaller particles have higher surface areas. In the adsorption study, large surface areas means the small particles are favored because more active sites are available for molecules that can attach to the surface of adsorbent^65^,^66^,^67^.

**Figure 7:**
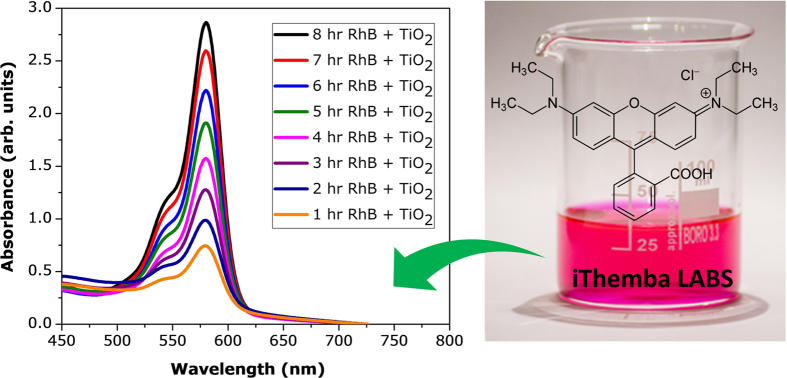
Photocatalytic studies of CeO_2_ doped TiO_2_ nanocomposites were synthesized hydrothermally at 700 °C for 9 hrs.

**Figure 8:**
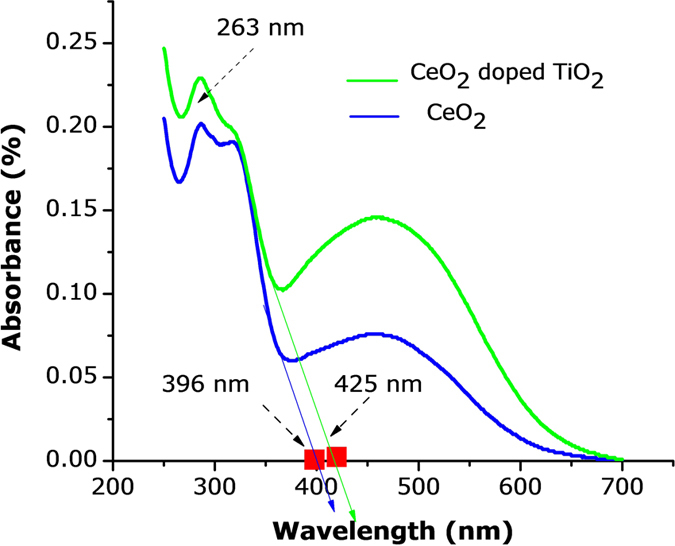
UV-is image of Ce-Ti-O_2_ nanocomposites were synthesized hydrothermally at 700 °C for 9 hrs.

The CeO_2_-doped TiO_2_ nanocrystals showed the potential for photocatalytic activity. The absorption spectra of RhB pink solution after 1 hr of UV-vis light irradiation in the presence of different CeO_2_-doped TiO_2_ nanocrystals is shown in Fig. 7. The percentage decomposition of the RhB pink absorption peak (578.21 nm) after 1 and 2 hrs was 93%. These results highlight the superior photocatalytic response of ceria-doped TiO_2_ nanocomposites^68^,^69^,^70^,^71^,^72^. The decrease in intensity of the absorption peaks, both in the visible and ultraviolet regions, with irradiation time indicated that the RhB was degraded. To compare the photocatalytic activity of CeO_2_-doped TiO_2_ nanocrystals, the absorption spectrum of the RhB solution after 2 hrs irradiation in the presence of TiO_2_ nanocrystals was also recorded. The percentage decomposition of RhB absorption peak after 8 hrs irradiation in the presence of CeO_2_-doped TiO_2_ photocatalysts increased to 99.89%. These results highlight that the addition of Ce improves the photocatalytic activity of TiO_2_ nanocrystals. The visible light photo activity of ceria-doped TiO_2_ can be explained by a new energy level produced in the band gap of TiO_2_ through the dispersion of ceria nanoparticles in the TiO_2_ matrix^73^,^74^. As shown in Fig. 7, an electron can be excited from the defect state to the TiO_2_ conduction band by a photon with energy equal to *hv*_2_. An additional benefit of transition metal doping is the improved trapping of electrons to inhibit electron-hole recombination during irradiation. The UV–visible absorption spectrum of CeO_2_-doped TiO_2_ nanocrystals is shown in Fig. 8. Although the wavelength of the Varian Cary 5E spectrometer was limited by the light source, the absorption band of the cerium oxide nanoparticles increased in wavelength because of quantum confinement of the excitons present in the samples compared with bulk cerium oxide particles.

The UV-vis spectrum showed strong absorptions below 263 and 396 nm, and a well-defined absorbance peak at around 425 nm. It revealed that the band gap of CeO_2_ nanocrystals was approximately 4.79 eV, which is greater than the value for the bulk CeO_2_ (E_g_ = 3.18 eV). As a result, quantum size effects will increase the bandgap leading to a blue shift in the absorption spectrum. Observing this optical phenomenon indicated that our nanoparticles showed the quantum size effect. The estimated bandgap energy (E_g_) of the samples was generated by substituting the obtained absorption edge (λ) values into the formula: E_g_ (eV) = 1260/λ (nm). As can be seen from Fig. 8, the absorption edges of Ce-Ti (425 nm) shifted to higher wavelength (red shift) compared to the absorption edges of Ti (396 nm), indicating a change of band gap (E_g_) from the presence of Ce in TiO_2_ host lattices. The E_g_ values obtained for Ce-Ti and pure Ce were 2.96 eV and 3.18 eV, respectively. Our photocatalytic results from the RhB dye decomposition in the presence of ceria-doped TiO_2_ nanocrystals resulted in a value of 2.17 eV. The absorption study revealed that the Ce-TiO_2_ nanocrystals were transparent in the visible region^75^,^76^,^77^. The absorption edge, determined from the peak maximum of the first derivative of the absorption plot, was at 263 nm (4.79 eV), which matches well with the standard bulk value band gap of Ce-Ti. Also, the steep rise of the absorption edge is an indication of the defect free structure of Ce-Ti nanocrystals^78^,^79^,^80^.

**Figure 9:**
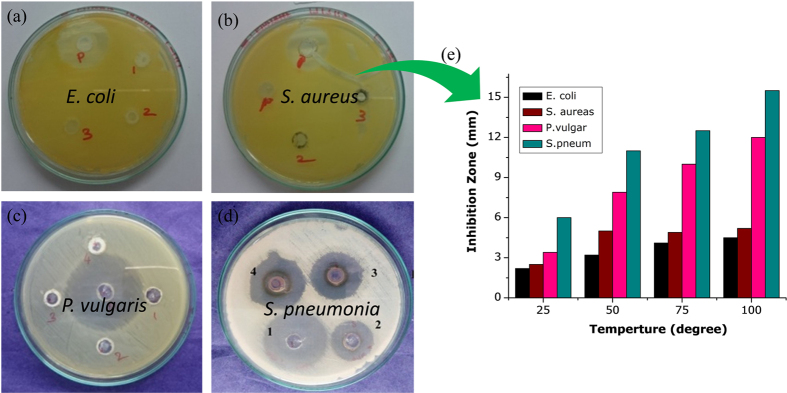
(a–e) Antibacterial performances of Ce-Ti-O_2_ nanocomposites were synthesized hydrothermally at 700 °C for 9 hrs.

The antibacterial activity of the ceria-doped TiO_2_ nanoparticles was investigated using the well diffusion method. Approximately 20 mL of sterile molten Mueller Hinton agar (Hi Media Laboratories Pvt. Limited, Mumbai, India) was poured into the sterile petriplates. Triplicate plates were swabbed with the overnight culture (108 cells/mL) of pathogenic bacteria *viz., E. coli, S. aureus, P. vulgaris and S. pneum*. The solid medium was gently punctured with the help of cork borer to make a well. Finally, the nanoparticle samples (50 μg/mL) were added from the stock into each well and incubated for 24 hrs at 37 ± 2 °C. After 24 hrs of incubation, the zone of inhibition was measured and expressed as a diameter in mm^81^. The antimicrobial properties of ceria-doped TiO_2_ nanocrystals are presented in Fig. 9(a–e). The zone of inhibition of both Gram positive and Gram negative microbial strains against these studied materials clearly confirmed that the activity was directly proportional to the concentration of CeO_2_-doped TiO_2_ nanocomposites. These results show that there is no inhibition on bacterial growth when the glass plate contained CeO_2_-doped TiO_2_ composites. Under UV light, a powder of CeO_2_-doped TiO_2_ nanocomposites was used to examine its effects on the antibacterial activity for negative and positive bacteria. Gradually the zone of inhibition of both pathogens increased with increasing doping concentrations of TiO_2_. Among the microbial strains, Gram positive strains were more susceptible to the studied compounds than Gram negative strains. Gram negative microbial strains have thick cell wall membranes and lip polysaccharides, and these substances stop the penetration of the nanocomposite material into the cells^82^,^83^,^84^.

In the minimum inhibitory concentration (MIC) study, different concentrations (5, 10, 25 and 50 μg/mL) of chosen nanoparticles were prepared with dimethyl sulfoxide (DMSO) and mixed with 100 μl/mL of nutrient broth and 50 μL of 24-hr old bacterial inoculum, and were allowed to grow overnight at 37 °C for 48 hrs. Nutrient broth alone served as a negative control. The whole setup in triplicate was incubated at 37 °C for 24 hrs. The MIC was the lowest concentration of the nanoparticles that did not permit any visible growth of bacteria during 24 hrs of incubation on the basis of turbidity. The minimum bactericidal concentration (MBC) to avoid the possibility of misinterpretation related to the turbidity of insoluble compounds, if any, was determined by sub-culturing the MIC serial dilutions after 24 hrs in nutrient agar plates using a 0.01 mL loop and were incubated at 37 °C for 24 hrs. The MBC was regarded as the lowest concentration that prevents the growth of a bacterial colony in this media.

## Conclusions

In summary, we report the synthesis and characterization of CeO_2_-doped TiO_2_ nanocrystalline composites prepared hydrothermally. These composites were well-suited for preparing ceria-based titanium oxides with high surface areas (approaching 100 m^2^g^−1^) and cation stoichiometries close to 1:1. The composites were characterized by regular crystallites with well-defined compositions and narrow size distributions that had a cubic phase structure. This result was likely because of the small crystallite size effect. All of the results showed that the prepared nanocomposites displayed higher surface redox reactivities than their parent single oxides prepared using the same technique. The moderate degree of compositional heterogeneity of the specimens obtained in this way (surface enrichment of Ce) was attributed to the different relative speeds of the precipitation of Ce and Ti. The percentage decomposition of the most intense absorption peak at 579.93 nm after 1 and 2 hrs of irradiation was 93%. After 8 hrs irradiation in the presence of CeO_2_-doped TiO_2_ photocatalysts this value increased to 99.89%. The zone of inhibition of both Gram positive and Gram negative microbial strains against these studied materials confirmed that the activity was directly proportional to the concentration of CeO_2_-doped TiO_2_ nanocomposites. Addition of Ce, as well as the nature of surfactant used, played an important role in reducing the crystallite size. The FTIR, PL and XPS analyses confirmed the ceria-doped titania nanocrystals formed at room temperature.

## Additional Information

**How to cite this article**: Kasinathan, K. *et al*. Photodegradation of organic pollutants RhB dye using UV simulated sunlight on ceria based TiO_2_ nanomaterials for antibacterial applications. *Sci. Rep.* **6**, 38064; doi: 10.1038/srep38064 (2016).

**Publisher's note:** Springer Nature remains neutral with regard to jurisdictional claims in published maps and institutional affiliations.

## Supplementary Material

Supplementary Information
