# Supplementary material for: Photodegradation of organic pollutants RhB dye using UV simulated sunlight on ceria based TiO2 nanomaterials for antibacterial applications
Source: Sci Rep. 2016 Nov 30;6:38064. doi: 10.1038/srep38064 (PMC5128916; doi:10.1038/srep38064)
Supplement: Supplementary Information [file srep38064-s1.pdf]

Photodegradation of organic pollutants RhB dye using UV simulated sunlight on ceria based TiO_2 nanomaterials for antibacterial applications

Kaviyarasu Kasinathan[1,2*], John Kennedy [1,3], Manikandan Elayaperumal[1,4], Mohamed Henini[1,5]
Maaza Malik[1,2]

[1]UNESCO-UNISA Africa Chair in Nanosciences/Nanotechnology Laboratories, College of Graduate Studies, University of South Africa (*UNISA*), Muckleneuk Ridge, P O Box 392, Pretoria, South Africa.

[2]Nanosciences African network (*NANOAFNET*), Materials Research Division (MRD), *i*Themba LABS-National Research Foundation (*NRF*), 1 Old Faure Road, 7129, P O Box 722, Somerset West, Western Cape Province, South Africa.

[3]National Isotope Centre, GNS Science, PO Box 31312, Lower Hutt 5010, New Zealand.

[4]Department of Physics, TVUAC, Thennangur, Thiruvalluvar University, Vellore, India.

[5]School of Physics and Astronomy, The University of Nottingham, University Park, Nottingham, Nottingham NG7 2RD, United Kingdom.

* kaviyarasuloyolacollege@gmail.com kavi@tlabs.ac.za

SUPPLEMENTARY FIGURES

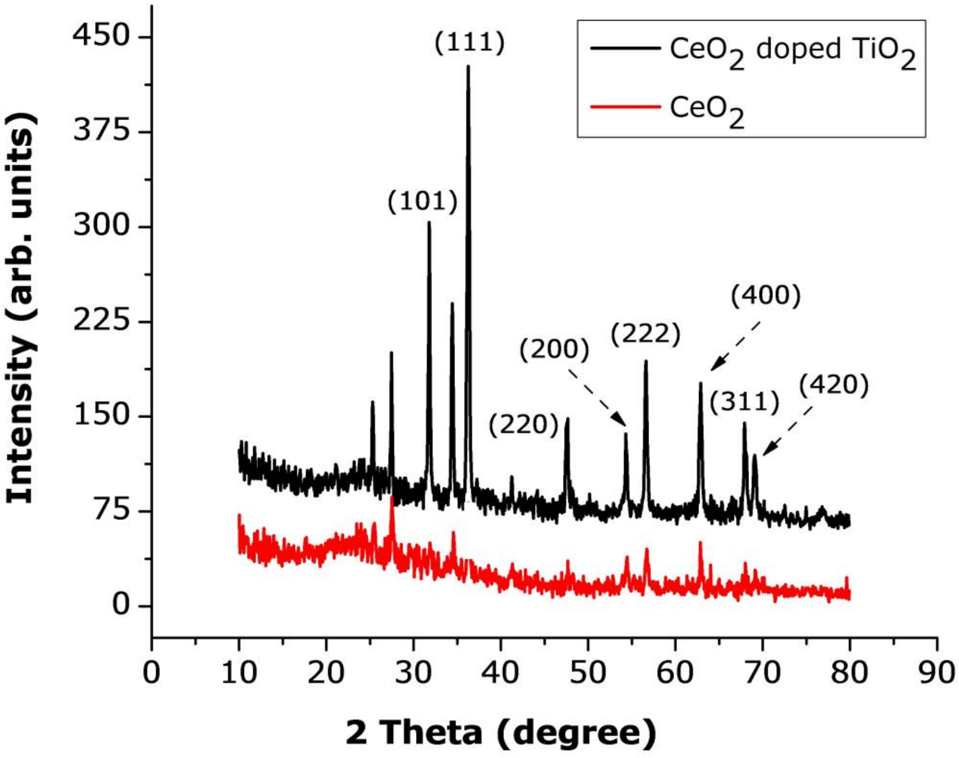

Supplementary Figure S1:- XRD pattern of ceria doped TiO_2 nanocomposites

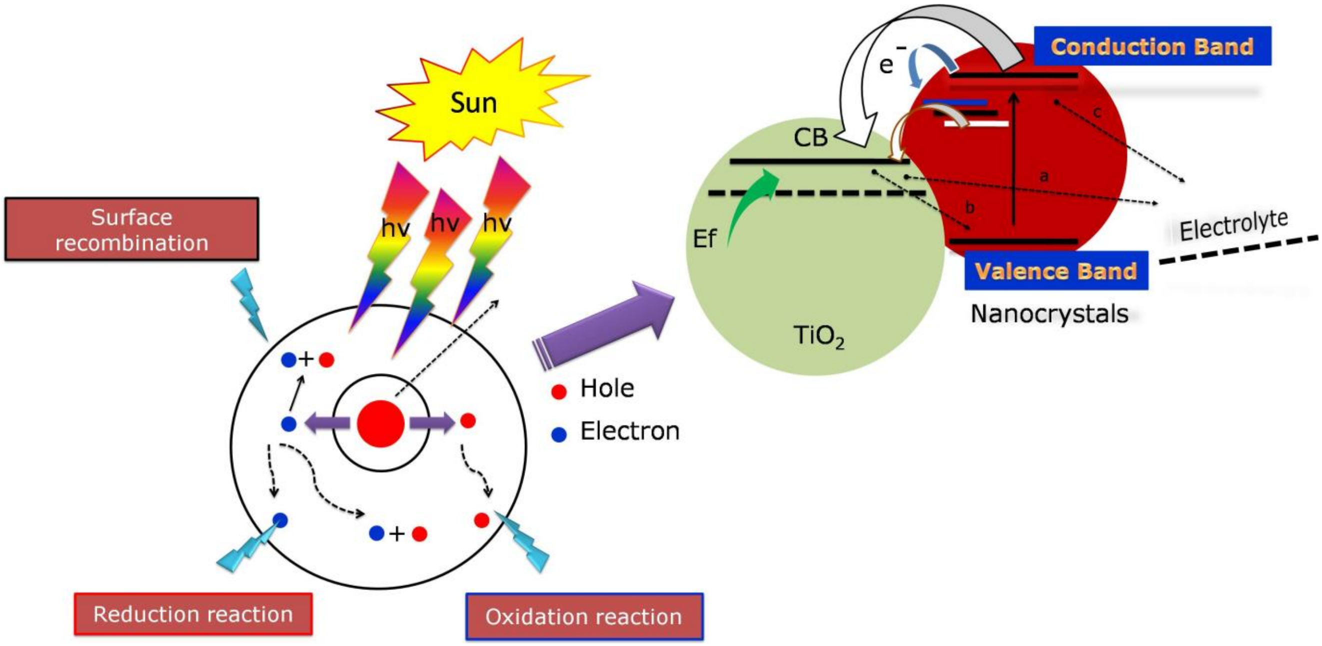

Supplementary Figure S2:- Schematic representation mechanism of photocatalytic of ceria doped TiO_2 nanocomposites

Supplementary Discussions

Figure S1:

The X-ray powder diffraction (XRD) experiments were measured on a RigaKu D/max-RB diffractometer with Ni-filtered graphite monochromatized Cukα radiation (λ = 1.54056 Å) under 40 kV, 30 mA and scanning between 10° to 70° (2θ). Structural characterization of the prepared sample is done using a X-ray diffractometer and CuK_{α} radiation (λ = 1.5418 Å) at room temperature. Intensity of the diffracted X-ray beam is recorded as a function of the angle 2θ. The X-ray diffraction pattern (XRD) of ceria doped TiO_2 nanocrystals as shown in supplementary figure 1. The cerium hydroxide decomposes into titanium oxide on heating upon heating the sample at 700 °C for 9 hrs. Because of the heating the color of the sample changes from yellow to black. Figure 1 shows the XRD pattern peaks associated with cubic Ce-TiO_2. Good agreement was found when comparing the interplanar distances (d_{hkl}) with the standard JCPDS data (No. 34-0394). The peaks corresponding to the 2θ values 27.23°, 36.49°, 47.58°, 54.14°, 56.51°, 62.69°, 67.61° and 69.48° are assigned to the Miller indices (101), (111), (220), (200), (222), (400), (311) and (420), respectively. The orientation of the nanocrystals is dominant along the (200) plane. The calculated unit cell parameter of the system (0.5326 nm) is perfectly matched with the standard value (0.5311 nm). It is evident from Figure 1 that annealing at 700 °C is necessary to obtain TiO_2 nanocomposites powder. The prominent peaks have been utilized the crystallite size can be estimated from the broadening of the peak measured in terms of full width at half maximum (FWHM). This is inversely proportional to the average crystallite size (D) as predicted by Scherrer's formula $D=0.94\lambda/\beta\cos\Theta$, where, β is the observed angular width at half of the maximum intensity of the peak given by $\beta_2=\beta'_2-\beta^0_2$, β' is the measured line width at half of the maximum and β^0 is the instrumental broadening which is 0.12° for the present study. From the analysis of positions and relative intensities of the diffracted peaks, the presence of single phase cubic structure of Ce-Ti-O with a (space group Fm_3m) is confirmed. However, one unidentified peak was observed at 2θ =36.23 in the CeO_2 nanocrystals which disappeared on calcination. The intensity ratio of peaks corresponding to (200) and (220) reflections of the synthesized nanosamples is less than 0.3 instead of 0.5 (ideal intensity ratio) suggests that the nanocrystals were grown in the specific crystallographic growth direction of [110]. The grain size estimated using the relative intensity peak (111) for cerium oxide nanocrystals was found to

be 22 nm and increase in sharpness of XRD peaks indicates that particles are in crystalline nature. Similar phenomenon was reported previously for 1D material. The (101), (111), (220), (200), (222), (400), (311) and (420) reflections are clearly seen and closely match the reference patterns for cerium oxide there is no impurity peaks were observed, revealing high purity of the sample. The calculated lattice constant based on the six distinct peaks is consistent, with standard data for (JCPD File No. 34-0394). On the other hand, the enhancement of crystallite size is very less with respect to the aggregated nanoparticles. The lattice parameter (*a*) of the as-synthesized nanocomposites was higher (0.5326 nm) than that of bulk CeO_2 (0.5311 nm) and it gradually decreased as the calcination temperature was increased and at 700 °C, it was 0.5315 nm. The lattice parameter of nanocrystalline CeO_2 is more than that of bulk due to the grain surface relaxation and presence of Ce^{3+}. This result is quite consistent for nanocomposites with literature reported results. Particles with size < 25 nm possess a cubic-like spherical structure. The properties of CeO_2 in the spherical are very close to that of doped TiO_2, whereas TiO_2 at surface tends to relax and the lattice parameter of surface CeO_2 increases locally. This grain surface relaxation contributes to the line broadening, thus tends to reduce the measured value of dislocation density. However, nanocrystalline CeO_2 have high redox capability due the existence of Ce^{3+} in high amount. In the XRD patterns of Ce-TiO_2 samples no additional peak for the corresponding titanium oxide was observed even after calcination at 700 °C, most probably due to the uniform dispersion of metal oxides which is beyond the identification level of XRD instrument. Surprisingly, the crystallite size of the Ce-TiO_2 nanocrystals is less than that of pure CeO_2 nanocrystals, but it is expected to increase due to further calcinations after impregnation [9]. However, the unit cell parameter of the titanium impregnated CeO_2 nanocrystals is less than that of pure CeO_2 nanocomposites. The reduction of the unit cell parameter evidenced that the titanium ion was incorporated in the crystal structure of CeO_2 nanocomposites during calcinations through diffusion and due to incorporation of small titanium ion (r_{Ti}^{2+} =0.087 nm) the unit cell parameter was reduced in agreement with reported rule. With the reduction of unit cell parameters, the crystallite size was also reduced for Ce-TiO_2 nanosamples.
